# Analysis of Gap Gene Regulation in a 3D Organism-Scale Model of the *Drosophila melanogaster* Embryo

**DOI:** 10.1371/journal.pone.0026797

**Published:** 2011-11-16

**Authors:** James B. Hengenius, Michael Gribskov, Ann E. Rundell, Charless C. Fowlkes, David M. Umulis

**Affiliations:** 1 Department of Biological Sciences, Purdue University, West Lafayette, Indiana, United States of America; 2 Department of Biomedical Engineering, Purdue University, West Lafayette, Indiana, United States of America; 3 Department of Computer Science, University of California Irvine, Irvine, California, United States of America; 4 Department of Agricultural and Biological Engineering, Purdue University, West Lafayette, Indiana, United States of America; Centre for Genomic Regulation (CRG), Universitat Pompeu Fabra, Spain

## Abstract

The axial bodyplan of *Drosophila melanogaster* is determined during a process called morphogenesis. Shortly after fertilization, maternal *bicoid* mRNA is translated into Bicoid (Bcd). This protein establishes a spatially graded morphogen distribution along the anterior-posterior (AP) axis of the embryo. Bcd initiates AP axis determination by triggering expression of gap genes that subsequently regulate each other's expression to form a precisely controlled spatial distribution of gene products. Reaction-diffusion models of gap gene expression on a 1D domain have previously been used to infer complex genetic regulatory network (GRN) interactions by optimizing model parameters with respect to 1D gap gene expression data. Here we construct a finite element reaction-diffusion model with a realistic 3D geometry fit to full 3D gap gene expression data. Though gap gene products exhibit dorsal-ventral asymmetries, we discover that previously inferred gap GRNs yield qualitatively correct AP distributions on the 3D domain only when DV-symmetric initial conditions are employed. Model patterning loses qualitative agreement with experimental data when we incorporate a realistic DV-asymmetric distribution of Bcd. Further, we find that geometry alone is insufficient to account for DV-asymmetries in the final gap gene distribution. Additional GRN optimization confirms that the 3D model remains sensitive to GRN parameter perturbations. Finally, we find that incorporation of 3D data in simulation and optimization does not constrain the search space or improve optimization results.

## Introduction

Embryonic development in *Drosophila melanogaster* is initiated with the formation of spatial morphogen distributions in the early embryo. The dynamic spatial patterns of diffusive morphogens encode information which specifies organism-scale development [1], [2]. Nonuniform initial spatial distributions of maternally deposited morphogen mRNAs, coupled with diffusion, decay, and complex genetic regulatory interactions, give rise to finer patterns that subdivide the dorsal-ventral (DV) [3]–[5] and anterior-posterior (AP) axes [2], [6] into distinct developmental regions.

**Figure 1:**
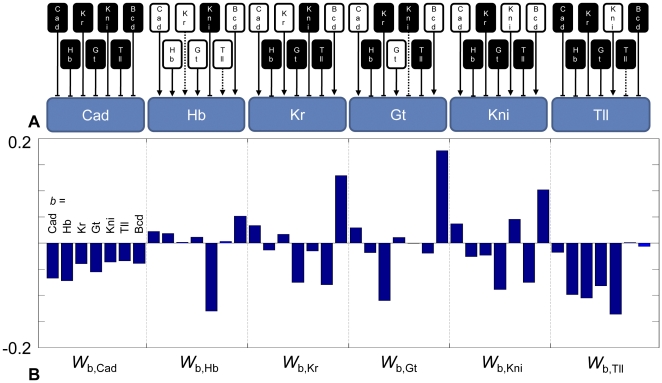
Gap gene genetic regulatory network. The model representation of the gap gene network. The network topology in (**A**) represents negative (black box, flat line) and positive (white box, arrowhead line) regulatory effects on each target gene (blue). Dashed lines represent near-zero regulatory inputs that may be negligible. This qualitative topology is quantified in (**B**) as a set of genetic regulatory network (GRN) weight parameters *w_b,a_*, the influence of gene b on gene a. From left to right, each set of seven inputs represent Cad, Gt, Hb, Kni, Kr, Tll, and Bcd. Each cluster of seven interactions represents a target gene Cad, Gt, Hb, Kni, Kr, and Tll.

The gap gene system is one of the most widely studied morphogen systems in *Drosophila* and is involved in delineation of boundaries of gene expression within the AP body plan [2]. AP patterning events begin approximately one hour post-fertilization. This patterning foreshadows the subsequent segmentation of the embryo [1], [2], [6]–[9]. During early development, the embryo is a polynucleated syncytium; most nuclei are arrayed in a thin layer near the surface of the embryo. Due in part to a cytoplasmic viscosity gradient common to insect embryos [10], morphogens (here, gap gene products) are thought to diffuse freely through periplasm near the embryonic surface and less substantially through the interior. Here, they regulate transcription within the periplasmic nuclei [2]. The process is initiated by the gene products of maternally-deposited, spatially-heterogeneous *bicoid* (Bcd), *caudal* (Cad), and *nanos* mRNAs [2], [11], [12]. Maternally deposited RNA species regulate expression of the gap genes: Hunchback (Hb, with a maternal mRNA contribution), Giant (Gt), Tailless (Tll), Krüppel (Kr), and Knirps (Kni) (see **Fig. 1a**) [11], [13], [14]. The gap genes, in turn, regulate the pair-rule genes which in turn control segment-polarity genes and embryonic segmentation [1], [2], [6], [15].

Most inferences regarding the gap genetic regulatory network (GRN) have been drawn from mutant and gene dosage studies in which the effects on morphology, gap, pair-rule, or segment polarity genes are observed [12], [16]–[36]. While these experiments are informative, it is difficult to unambiguously derive genetic regulatory interactions from such data; phenotypic changes may arise via direct action of the perturbed gene or via downstream targets of that gene. In contrast, Reinitz, Jaeger, and others applied a reverse engineering approach using dynamic wild-type data. Computational studies have modeled gap gene patterning using 1D partial differential equation (PDE) systems or ordinary differential equation systems that include an implicit approximation to the PDE [13], [14], [37]–[40] and logical rule sets [41]. These models represent the lateral trunk region of the *Drosophila* embryo along the AP axis, typically omitting the anterior and posterior end regions (with the exception of [40]). GRN topology is represented by a regulatory weight matrix and gene expression is modeled by a transfer function that sums the regulatory impact of each regulatory protein on expression of the others (see **Fig. 1b**) [13]. Model-driven inferences about GRN topology (i.e., inferring whether and to what degree one morphogen regulates expression of other morphogens) have been obtained by inverse modeling: optimizing the regulatory weight matrix against experimental gap gene expression data in hopes of recovering “true” GRNs [14], [42]–[45]. Findings have been mixed. Biological systems are thought to be robust (and thus insensitive) to perturbations. Some GRN parameters are highly sensitive while considerable uncertainty is associated with others [44], [45].

Previous 1D PDE models have been used effectively to infer network topology and investigate patterning regulation [13], [14], [42], [45], [46], but there are some questions that are better investigated using a full 3D spatial patterning model. Many important 3D effects, including variable diffusive path lengths around the embryo surface and optimization against 3D data, cannot be observed in a 1D model domain. DV asymmetries in gap gene distribution and possible interactions between the gap gene system and DV patterning systems are also neglected. Further, these 3D data may serve to constrain GRN optimization and inference.

Quantitative spatiotemporal atlases of gene expression data in the *Drosophila* embryo have been published and provide the starting point for quantitative analysis. [47]–[49]. The atlas includes measurement of gap gene expression collected from hundreds of individual embryos and registered onto a standardized 3D mesh of nuclei coordinates using pair-rule gene expression patterns as fiduciary points (mesh coordinates available in File S1). This composite VirtualEmbryo (VE) is a logical starting point for the development of 3D embryonic GRN models. It provides a ready-made embryonic geometry for full spatial PDE representations of the gap gene system. It also contains quantitative expression data against which we can optimize model parameters (and thus infer GRNs).

Using the VE data, we evaluate the impact of 1D model assumptions, conversion from 1D to 3D geometries, and incorporation of fully 3D protein distribution data in model simulation. Herein we reconstruct the 1D gap gene model of Jaeger *et al.* [13] using the finite element method (FEM) and extend it to the 3D VE geometry (**Fig. S1**). The 1D model of Jaeger *et al.* [13], 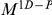 (see Table 1 for model definitions), is refit to lateral expression data from the VE. We then extend the 1D model PDEs to the full 3D embryonic geometry described by Fowlkes *et al.* and compare GRNs inferred from 1D and 3D models. Though 1D models focus on the lateral AP axis in 1D simulations, gap genes are not uniformly distributed along the DV axis. Coupled with the 3D geometry, DV asymmetries in initial conditions may encode positional information partially responsible for the observed AP patterning. As a preliminary exploration of asymmetric DV effects in an embryonic geometry, we evaluate the model using DV-asymmetric Bcd concentration data from thirteen embryos compiled in the VE.

**Table 1:** Model Variants and Corresponding Optimal Parameter Sets.

Model	Geometry	Initial Conditions	Optimal GRN Parameters* ( 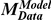 )
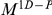	1D domain representing partial 35%–92% AP axis	Gt_0_ = Kni_0_ = Kr_0_ = Tll_0_ = 0; Bcd_SS_, Hb_0_, Cad_0_ values in Jaeger *et al.* [13]	*N/A* (evaluated with Jaeger *et al*.'s reported GRN, 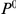 )
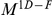	1D domain representing full 0%–100% AP axis	Gt_0_ = Kni_0_ = Kr_0_ = Tll_0_ = 0; Bcd_SS_, Hb_0_, Cad_0_ values in Jaeger *et al.*	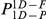 (fit to Jaeger's model output); 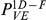 (fit to VirtualEmbryo data)
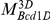	VE 3D domain	Gt_0_ = Kni_0_ = Kr_0_ = Tll_0_ = 0; Bcd_SS_, Hb_0_, Cad_0_ values in Jaeger *et al.* projected about AP axis (see **Fig. 3b–d**)	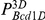 (evaluated with GRN 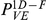 )
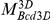	VE 3D domain	Gt_0_ = Kni_0_ = Kr_0_ = Tll_0_ = 0 Hb_0,_ Cad_0_ values in Jaeger *et al.* projected about AP axis. Bcd_SS_ interpolated from VE data (**Fig. 3e**).	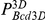 (evaluated with GRN 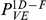 )
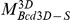	VE 3D domain	Gt_0_ = Kni_0_ = Kr_0_ = Tll_0_ = 0; Hb_0,_ Cad_0_ values in Jaeger *et al.* projected about AP axis. [Bcd]_SS_ interpolated from VE data and smoothed (**Fig. 3f**).	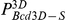

*optimized by fitting model output to Virtual Embryo data unless otherwise noted.

In addition to GRN sensitivities highlighted by previous 1D analyses [14], [38], [39], [44], [50], we find that the 3D model exhibits fragility with respect to the shape of maternal gradients: GRNs which were inferred by optimization of 1D models showed similar gap gene patterning when applied to 3D models with DV-symmetric Bcd. However, these GRNs gave rise to qualitatively different patterns in DV-asymmetric models. These realistic Bcd gradient models also captured some of the DV-asymmetries in gap gene patterning. The 3D models were also sensitive to small perturbations in GRNs; regulatory networks which were qualitatively similar (i.e., all network interactions maintained the same excitatory or inhibitory relationships and differed only by small changes in magnitude) led to qualitatively different gap gene patterns. Refitting of the DV-asymmetric 3D model to VE data produced a GRN which was similar to 1D GRNs but which produced an improved fit.

Another question addressed in this study is whether inclusion of 3D data improves optimization by the inclusion of additional constraints without increasing the degrees of freedom in the model. Unexpectedly, we found that the incorporation of additional 3D information in the form of a realistic DV-asymmetric Bcd worsened the error between optimized 3D models and data. This suggests the involvement of additional regulators in the formation of DV-asymmetries and indicates a direction for future modeling studies.

## Results

### One-Dimensional Model Analysis

**Figure 2:**
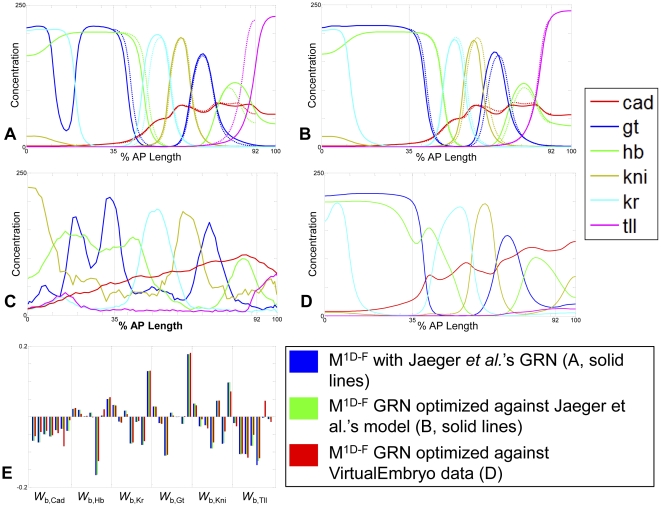
One-dimensional model results. Model output was simulated over a 0–100% AP length domain using the optimal GRN reported by Jaeger *et al*. Solid vertical lines represent the original model boundaries, not used in this simulation. (**A**) 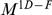 (solid lines) shows qualitative agreement with the Jaeger model 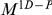 (dashed lines) in the 35–92% AP range, but shows discrepancies at either end of the domain due to the movement of boundaries; all species displayed at *t* = 70 min. (**B**) The best-fit GRN from Jaeger et al. was locally optimized to improve the agreement of the 0–100% AP length, model 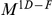 (solid lines), and the original Jaeger *et al*. original model (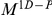 dashed lines); all species displayed at *t* = 70 min. (**C**) VE protein data for Gt, Hb, Kni, Kr at *t* = 70 min; VE mRNA data for Tll at *t* = 70 min; protein data from Jaeger *et al*. for Cad at *t* = 56 min. (**D**) Model output (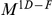) was also optimized against VE data (RMSE = 13.992); Gt, Hb, Kni, Kr, Tll at *t* = 70 min; Cad at *t* = 56 min. Despite modest improvements in model agreement in the 35% and 92% region (**C–D**), the resulting changes in parameter values were small. (**E**) Optimized parameter magnitudes vary but signs remained the same in most cases (blue - 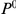; green - 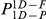; red - 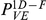).

Before analyzing the effects of embryonic geometry and DV-asymmetric positional information, we reimplemented the 1D model of Jaeger *et al.* using the finite element method. In this work we denote model variants with *M*; superscripts represent model domains and subscripts signify initial conditions if multiple initial conditions are used. The 1D model of Jaeger *et al.* is called 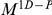 (using a 1D domain representing a partial AP length of 35%–92%; full model nomenclature available in **Table 1**). We verified 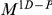 against Jaeger *et al*. 's simulated output. Whereas the original model limited gene expression to a finite number of discrete nuclear coordinates along the 35–92% region of the embryonic AP axis, the FEM approximates a continuous solution to these equations along this domain. Discrete versus continuous model comparisons by Gursky *et al.* suggest that embryonic patterning is not strongly coupled to nuclear position and that continuous models are comparable to discrete models of gene expression [51]. Our results agree with this finding. FEM simulations produce model output comparable to Jaeger *et al.* 's discrete 1D model (**Fig. 2a**, dashed line, cf. Figure S20 in [13]).

Though 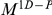 recapitulated previous results when simulated in the region from 35–92% on the AP axis, we sought to determine whether moving the boundaries to the embryo ends perturbed gap gene patterning in the trunk region. It is unclear *a priori* how modification of boundary conditions might impact the model output, because the selection of boundaries at 35% and 92% in earlier work appears to coincide with either maxima or minima of gap gene distributions; at these positions, spatial derivatives are near zero and diffusive flux may be negligible. Using no-flux boundaries at 0% and 100% EL, coupled with the parameters and initial conditions specified in the original model [13], we evaluated 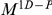 and 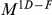 using the GRN parameters 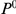 reported by Jaeger *et al*. [13]. Herein, parameter sets are denoted *P* and super- and subscripts have model-specific meanings. The simulated patterns from the original 35–92% AP and the 0–100% AP domains are shown in **Figure 2a**'s dashed and solid lines, respectively.

Pronounced shifts in Tll and Kr distributions, coupled with the qualitative change in the anterior Gt distribution, demonstrate the role boundary conditions play in the in the distribution of gap gen products for a given set of parameters. Though the output of 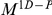 qualitatively resembles the expression data collected previously when evaluated at 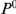 [13], these findings suggest that 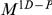
's agreement with data arises from a combination of the inferred GRN and the domain's boundary conditions. Thus, the internal zero-flux boundary conditions used in previous models may bias GRN inference. To evaluate the impact of boundary placement on GRN inference, we performed a numerical gradient descent search of the parameter space to minimize the root mean squared error (RMSE) between 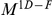 and 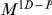 (represented by the dashed line in **Figure 2a**). The search was initialized with the previously reported optimal 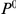. The result of this search, optimized GRN 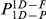 (superscript denotes the model being optimized and subscript denotes data with which the model is optimized), is illustrated in **Figure 2b**. Here, the output of 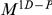 represents extant models' with internal no-flux boundaries.

Though domain boundary placement affects the banding pattern, **Figure 2b** suggests that these constraints have a limited effect on GRN inference. Optimizing the GRN parameters of 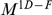 to fit the original model output recovered a quantitatively similar patterning within the 35–92% AP length of the full 1D domain. Additionally, the optimized GRN 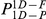 was qualitatively similar to 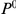 (e.g., though optimized parameters underwent small changes in magnitude, all parameters maintained the same sign, **Fig. 2e**).

To facilitate a direct comparison between 1D and 3D models presented herein, we first evaluated the goodness-of-fit between the 35–92% AP (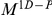) and full AP domain (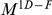) 1D models using VE data. When possible, we use protein expression data from the VE: Gt, Hb, Kni, and Kr protein data is available across six equidistant time points spanning 50 minutes. Tll protein data is unavailable and we use Tll mRNA data as a surrogate for the protein distributions. Cad protein distributions are also unavailable in the VE; we substitute 1D Cad data from Jaeger *et al*. [13] that spans 45 minutes with seven time points. Because the 1D model domains represent the lateral region of the full embryo, we extracted expression data from this region of the VE (**Fig. 2c**). We performed a constrained search of GRN parameters initialized at 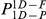 to yield an optimized GRN 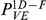 (subscript *VE* denotes VE training data). The resulting model output and a comparison of model parameters are shown in **Figures 2d–e**.

Though 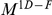 was capable of recovering the output of 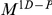 (with parameter set 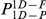) and VE data (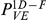) within the 35–92% AP axis, poor fit to VE data persisted outside of this region. The 0–35% and 92–100% AP regions exhibit qualitative disagreement with VE data in these regions consistent with the biological requirement for additional head and tail patterning genes (**Fig. 2c–d**).

### Three-Dimensional Model Analysis

Beginning with the GRN optimized on the full 1D domain, we extended the model to a 3D domain using the geometry in the VE. This was performed by implementing the system of PDEs on a 2D surface “wrapped” around the VE geometry. We used this model to evaluate the effects of both model geometry and DV-asymmetric initial conditions on model output.

**Figure 3:**
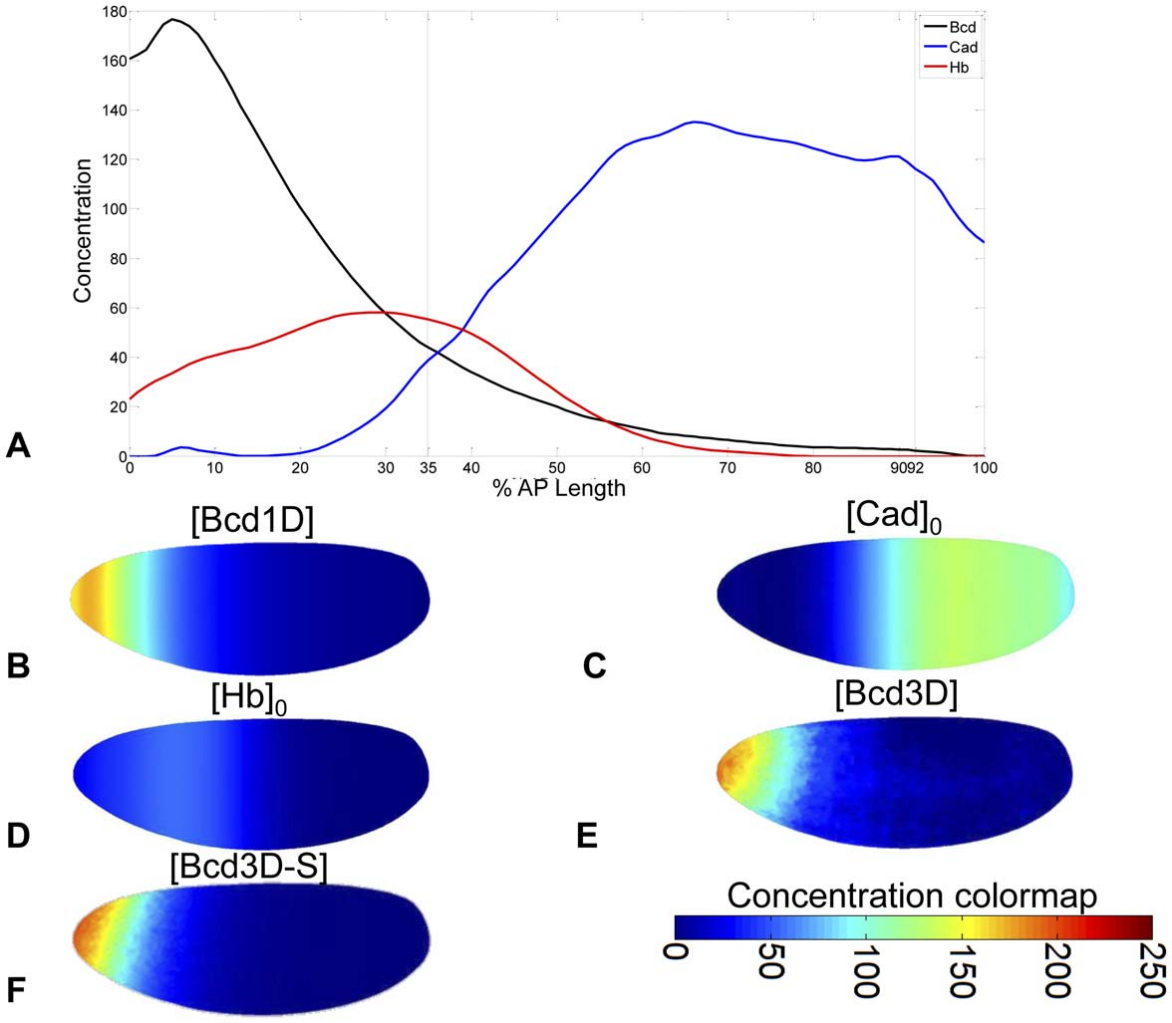
1D and 3D initial conditions. Initial conditions in various models. (**A**) 1D model initial conditions, reported by Jaeger *et al*., and used in models 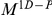 and 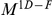. (**B**) 1D initial conditions were mapped onto the 3D embryonic geometry (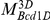). (**C**), 1D initial Cad protein distribution, (**D**) 1D initial Hb protein distribution. Subsequent models incorporated (**E**) DV-asymmetric interpolated [Bcd] distribution (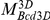) or (**F**) smoothed DV-asymmetric interpolated [Bcd] distribution (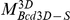).

**Figure 4:**
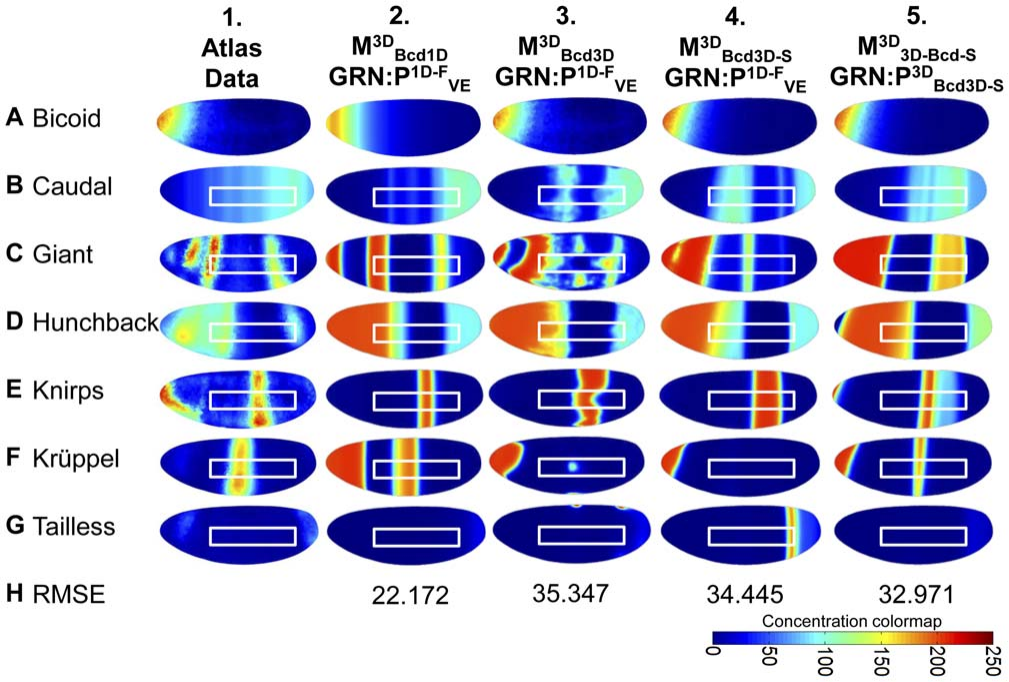
Three-dimensional model results. Simulation results in the 3D model. (**A–H**) Lateral view of VE geometry is shown in rows **A–G** (Gt, Hb, Kni, Kr, Tll at *t* = 70 min, Cad at *t* = 56 min); row **H** displays RMSE difference between model and VE data summed with all time points. Column 1 shows scaled VE data. Column 2 displays output from 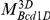 evaluated with GRN 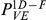. Column 3 contains output from 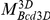 incorporating DV-asymmetric Bcd data and GRN 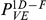; Column 4 illustrates the effect of the smoothed Bcd interpolant in 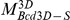 while considering the same GRN 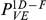. Column 5 displays output from 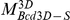 with reoptimized parameters 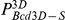. White boxes indicate the lateral areas where Jaeger *et al*. optimized their 1D model. Animations of pattern development are available for column 2 (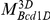 , **Movies S1, S2, S3, S4, S5, S6**) and column 5 (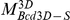, **Movies S7, S8, S9, S10, S11, S12**) in the supplementary material.

To assess the effects of model geometry on patterning independent of initial conditions, the model was first simulated using DV-symmetric initial conditions (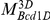): Bcd, Hb, and Cad distributions at time zero were obtained from the original 1D model and projected around the surface of the embryo (**Fig. 3a–d**). Evaluated at the previously inferred optimal 1D GRN (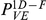), model 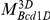 yielded patterning qualitatively similar to the full length 1D model output (**Fig. 4a–g** **, column 2**). To confirm our derivation of the diffusion constants (see Methods) and rule out unintentional adjustment of the diffusive length constant (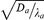), we performed a continuation of diffusion constants while holding decay parameters (*λ_a_*) values constant (**Figs. S2, S3**). While band overlap does vary with diffusion constants, they are quantitatively similar. Interestingly, symmetric Bcd models appear robust against increased diffusion (**Fig. S2**) while increased diffusion disrupted patterning in asymmetric Bcd models (**Fig. S3**). The pattern formation timecourse for Bcd-symmetric patterning is animated in **Movies S1, S2, S3, S4, S5, S6**.

Though there are some DV-asymmetries present in the output (e.g., slight curvature of the anterior Gt stripe), 1D versus 3D domain geometry alone has only a modest impact on DV patterning of gap genes. This suggests that the pronounced DV-asymmetries present in the final distributions of the proteins at the onset of nuclear division 14 (**Fig. 4** **, column 1**) stem from other sources. We consider the effect of spatial information encoded in initial DV asymmetries of protein distributions. The coupling of gap gene regulation with DV-patterning systems [5], [52], [53] is another possibility.

### Effect of Dorsal-Ventral Asymmetric Bcd

To evaluate the impact of DV-asymmetric inputs on the model, we modified the steady-state Bcd distribution shown in **Figure 3b** to incorporate a realistic DV gradient (**Fig. 3e**). Unlike other morphogens, the Bcd distribution is static over the entire time course of model simulation. This allowed us to create a single interpolant of VE Bcd data and use it as a model input for all 70 minutes of the simulation. The pattern formation timecourse for Bcd-asymmetric patterning is animated in **Movies S7, S8, S9, S10, S11, S12**.

Evaluated at the optimal 1D GRN 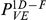, model 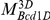 produces patterning that is radically different from DV-symmetric 1D (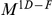) and 3D (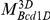) models (**Figs. 4a–g** **, column 3**). The most striking example of this is the Kr model output; whereas Kr forms a full band *in vivo*, 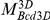 lacks full lateral expression of Kr and has an anomalous region of expression at the anterior end of the embryo (**Fig. 4f** **, column 3**). Similarly, the simulated Hb concentrations remain above observed levels (**Fig. 4d** **, column 3**). The posterior Hb band also shifts to the posterior end of the embryo. Gt exhibits qualitative disagreement with the VE data; whereas anterior Gt expression is observed only in a limited dorsal region of the embryo (**Fig. 4c** **, column 1**), the anterior of the 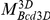 is saturated with Gt (**Fig. 4c** **, column 3**). Further, though the experimentally observed posterior Gt band (**Fig. 4c** **, column 1**) is predicted by simulation, it exhibits unusual differences in width along the DV axis. As in previous versions of the model, the best agreement between model and data was found in the lateral 35–92% AP region (**Fig. 4b–g** **, column 3 white boxes**).

The cell-to-cell variability in patterning found for many simulated proteins (e.g., Gt, Cad, and Kni) in 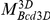 led us to consider the effect of noise in the VE Bcd distribution. Diffusion of Bcd may serve to smooth this variation *in vivo*; our use of a single static Bcd interpolant in 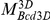 leads to an artificial persistence of the noise found in VE data (**Fig. 3e**). To test for and remove this artificial condition, we created a regularized version of the Bcd interpolant (**Fig. 3f**). This was constructed by building a simple source diffusion decay (SDD) reaction-diffusion model of Bcd alone [18]. This SDD model was fit to VE data and the steady-state solution was used as the smoothed Bcd interpolant. The model incorporating regularized Bcd, 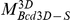, did not show significant improvement over 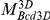 when evaluated with 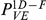 (**Fig. 4 a–g** **, column 4**). However, it did eliminate the cell-to-cell variability present in 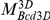. The model's artificial sensitivity to Bcd noise was especially evident in Gt (**Fig. 4c** **, columns 3–4**). Two anterior and one posterior Gt bands in 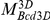 changed width and AP position after smoothing of Bcd. This result suggests that while diffusion may serve as a buffer against transient stochastic variations in protein expression and local concentration (in agreement with stochastic simulation [54]), sustained cell-to-cell variability has the potential to disrupt patterning.

Having observed that a GRN inferred on the 1D domain (and lacking DV asymmetries) produces a qualitatively incorrect fit compared to 3D data, we attempted to optimize the GRN with Matlab's constrained search function fmincon() initialized at 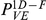 (previously used to estimate 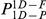 and 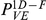). This approach failed to reduce model error. Fomekong-Nanfack *et al.* demonstrated that 1D gap gene systems are amenable to optimization by evolutionary algorithms [45]. We therefore employed a genetic algorithm (GA) to more broadly survey the parameter space. Do to computational cost, we used a small population size of 20 genomes to search the GRN parameter space (42 parameters), the GA identified an optimal GRN for 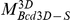. The resulting GRN, 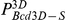, led to a reduction in model error and a modest qualitative improvement with respect to 3D data (**Fig. 4** **, column 5**). The lateral Kr band missing from the 1D-inferred GRN 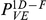 (**Fig. 4f** **, columns 3–4**) is restored (**Fig. 4f** **, column 5**), though it is not as wide as the experimentally observed band. Tll no longer shows relative over-expression at the posterior end of the embryo (**Fig. 4g** **, column 5**). Hb continues to exhibit relative over-expression at the anterior end of the embryo, though its posterior band is shifted closer to its correct position (**Fig. 4d** **, column 5**). Similarly, the anterior distribution of Gt extends beyond the dorsal region observed in the VE (**Fig. 4c** **, column 1**). However, its posterior band is now located correctly in **Figure 4c** **, column 5** (though it is wider than the observed protein band). Beyond differences in concentration of individual proteins, DV-asymmetric Bcd causes a notable qualitative difference in the AP position and emergence of protein bands. Compared to the 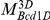 (**Fig. 4** **, column 2**), the DV-asymmetric GRNs (**Fig. 4** **, columns 4–5**) exhibit DV-asymmetries in their output. For example, the dorsal terminus of the anterior Gt band is posterior to its ventral terminus; it is splayed toward the anterior. This behavior agrees with observed data in the anterior half of the embryo, but the expected DV curvature is either absent (posterior Hb **Fig. 4d** **, column 5**) or inverted in the posterior half of the embryo. For example, Kni, whose dorsal terminus should exhibit posterior-splaying (**Fig. 4e** **, column 5**), is inverted. This DV curvature corresponds in direction to the DV asymmetry of Bcd. The absence of reversed splaying in the output in the posterior portion of the model (though present in the data) suggests that the model may be lacking additional posterior determinant(s) affecting the gap gene system.

**Figure 5:**
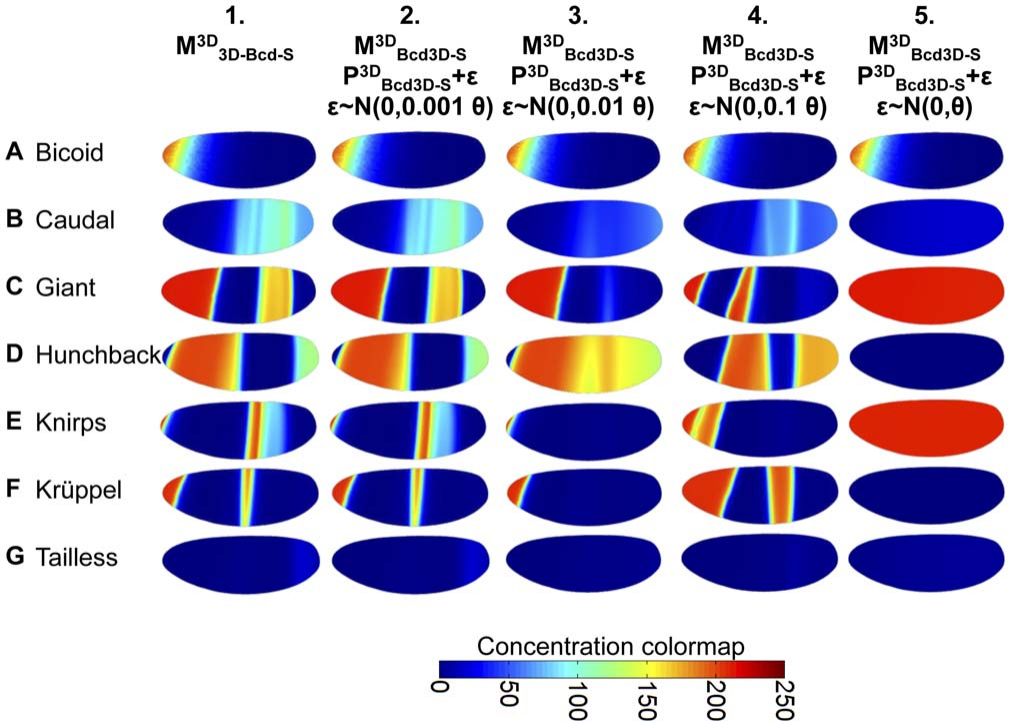
Model 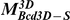 is not robust to noise in GRN parameters. Parametric noise alters model output. Lateral view of VE geometry for all genes is shown in rows **A–G** (all outputs at *t* = 70 min). Each column displays 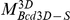 output at t = 70 min evaluated with GRN 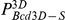. Columns 2–5 represent randomly chosen sample output when a normally distributed noise vector ε is added to the GRN parameter set (denoted θ). ε has mean of 0 and variance that scales with θ.

In the 3D regime, 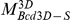 demonstrated considerable sensitivity to small changes in GRN parameter values. The model was simulated after adding normally distributed noise scaled by each parameter value, *p_i_*, across a range of magnitudes (sample model output in **Fig. 5**). The model gives output qualitatively similar to the optimal GRN 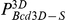 only when parameter noise is low (e.g., 0.1% *p_i_* in **Fig. 5** **, column 1**). All other simulations, with noise terms of 1%*p_i_* and higher, yielded drastically and qualitatively different outputs.

>In summary, the GRNs we inferred in this study are qualitatively similar: magnitudes of parameters vary by approximately 10% and parameter sign stays the same in all but a few low-magnitude parameters (see **Table S1**). A notable exception is the regulatory parameter for the Kni→Tll interaction; here the sign of the parameter (and thus the regulatory relationship) is reversed. However, we acknowledge that the treatment of Tll as a state variable under gap gene regulation is artificial and this biological relevance of this observation is questionable. Optimization leaves most regulatory parameters with the same sign and changes only the magnitudes, and those regulatory weights which change sign have small magnitudes (i.e., small regulatory effects). The use of a global search method (GA) to optimize 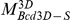 did not recover a superior GRN that differed qualitatively from the original *P* ^0^.

## Discussion

The understanding of *Drosophila* developmental gene regulation has benefited from advances in quantitative modeling of gene regulation. However, existing PDE models of AP patterning have been limited to 1D approximations of the 3D geometry. By extending a model of gap gene regulation to a 3D embryonic geometry and adding realistic DV-asymmetry to upstream maternal Bcd, this work allows us to pose new questions about the effects of embryonic shape and DV gradients on gap gene patterning. Jaeger *et al*. 's 2004 model has been succeeded by more recent models of gap gene development incorporating additional regulatory inputs [37]–[39], [46], [55]–[58]. However, recent models of AP patterning retain partial domains (e.g., 35%–92% AP) with internal no-flux boundary conditions and use regulatory schema similar to **eqns 1** **–** **3** (see **Methods**) to represent GRNs. We chose the Jaeger *et al*. 's 2004 model as a case study in 1D vs. 3D modeling because it is the representative of many existing 1D models.

Before comparing 1D and 3D geometries, we examined the effect of boundary position in PDE solutions. Though embryos do not contain physical barriers to diffusion at 35% and 92% of the AP axis, small spatial gradients (**Fig. 2a** **, dashed lines**) at those positions suggested that small diffusive flux would minimize the effects of these internal boundaries. However, we found that the system was sensitive to boundary placement (cf. **Fig. 2a** **, solid lines**). Though this finding indicates the importance of using biologically realistic boundary conditions (i.e., no-flux boundaries at 0% and 100% AP), the simulations in **Figure 2** also illustrate our limited representation of regulation beyond the 35%–92% trunk region: Omission of terminal gap genes and regulators result in optimized parameter sets that cannot recapitulate expression patterns from 0%–35% and 92%–100% AP in 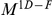 (**Fig. 2a,c**). Optimization to correct the boundary artifacts (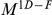 with 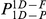) likewise fail to improve agreement with data outside of the 35%–92% region (**Fig. 2b**). The inclusion of terminal gap genes such as Huckebein in 1D gap gene models [37] provides a basis for extension to full 100% AP 1D and 3D models, though inclusion of Huckebein in a recent 3D modeling study yielded only modest improvements in overall cost and qualitative agreement at the AP extrema [59].

Prior analyses demonstrated the sensitivity of gap gene models to GRN parameter values [14], [43], [44] and examination of boundary conditions support this finding: GRN parameter optimization corrected boundary artifacts with extremely small changes to parameter values (**Fig. 2e**). Optimization against VE data produced similar small changes in GRN parameters (**Fig. 2e**). The GRN sensitivity of 1D models 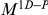 and 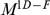 was also found in 3D models. **Table S1** collects all parameter values and reports the standard deviation for each parameter across 1D and 3D model optimizations. Parameter *w_Gt,Bcd_* exhibits the highest deviation across models with a standard deviation of 0.05, but this represents only 13% of the total parameter range ([−0.2,0.2]). These small changes in GRN parameters do more than shift protein band location as observed in **Figure 2**; they are capable of effecting qualitative patterning changes (e.g., changing the number of protein bands present on the embryo). For example, the transition from 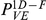 to 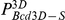 in model 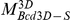 leads to the loss of a posterior Gt band and the creation of a posterior Kr band (**Fig. 4c,f** **, columns 4–5**). **Figure 5** shows randomly selected sample model outputs at *t* = 70 min with increasing levels of normally distributed noised added to the GRN parameter vector. One percent noise was sufficient to induce qualitatively different banding patterns on the 3D geometry. The qualitative changes in patterning for all but the smallest levels of noise confirm the observations of parameter sensitivity in 1D and 3D models. The extreme sensitivity of model outputs to small changes in GRN parameters challenges analyses of GRN evolution positing phenotypically robust fitness landscapes [60]–[62]. Unfortunately, the computational expense of PDE models prevented an exhaustive exploration of the GRN parameter space and corresponding approximation of a fitness landscape. The fragility of the gap gene system to GRN perturbations bears further study, especially in its contrast to prevailing thoughts that evolution occurs on networks with highly-connected neutral (selectively equivalent) genotypes.

In addition to the parameter sensitivity and boundary conditions, our work also demonstrate the use of accurate 3D geometry and its effects on model predictions. We found that geometry alone has a limited effect on gap gene patterning: Excepting slight DV-asymmetry brought about by the curvature of the 3D embryo, 1D output from 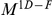 (**Fig. 2d**) and 3D output from 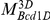 (**Fig. 4** **, column 2**) display qualitatively similar band position along the AP axis. The path length from anterior to posterior extrema differs with DV position: For example, the distance from anterior to posterior extrema is shorter along the dorsal surface than the ventral surface. We thought that this difference in diffusion distance might account for the anterior splaying displayed in VE data (**Fig. 4** **, column 1**), but this was not the case.

Though the 3D embryonic geometry was insufficient to explain DV-asymmetries in gap gene data, it allowed us to explore the effect of DV-asymmetric protein distributions on patterning. Notably, the 1D Bcd distribution of 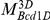 (**Fig. 3b**) differed from the typical dorsal-anterior distribution [63], [64] also found in the VE (**Fig. 3e**). Experimental noise in this data led to aberrant patterning in most gap genes in 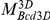 (**Fig. 4** **, column 3**), but a regularized version of the distribution (**Fig. 3f**) produced cleaner (though qualitatively incorrect) band appearance and position in 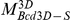 (**Fig. 4** **, column 4**). It also produced anterior-splaying in the anterior bands of Gt, Hb, Kni, and Kr. As previously noted, optimization of the sensitive GRN parameters improved qualitative agreement in model patterning with only small changes to parameter values (**Table S1**).

When considering 3D models and the data associated with them, we endeavored to identify any constraints on model optimization. This model has many degrees of freedom and additional information encoded in the DV asymmetries of gap genes might better guide parameter searches toward accurate GRNs. However, we observed no improvement in RMSE values and failed to find any novel GRNs for DV-asymmetric models.

Though our ensemble of models has led to interesting findings, we acknowledge model limitations. Recent modeling studies recognize that Cad and Tll patterning cannot be completely accounted for by gap genes in existing models; maternal mRNA complicates Cad expression and Tll is under the regulation of additional proteins [38]. Instead, newer models use data interpolants to represent these proteins [38]. The absence of these interpolants in our models may contribute to the unrealistic sensitivity of the 3D model parameters and DV-information. 3D interpolating functions incorporating VE data for Cad and Tll are under development; we will use these to explore the behavior of more recent 1D models on the 3D embryonic geometry.

The primary focus of this work is the comparison of 1D and 3D model geometries. **Figures 2d** and **4** **, column 2** reveal that differences in model geometry can be accommodated by relatively minor adjustments to GRN parameters. The 3D implementation (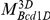) exhibits minor DV-asymmetries but otherwise mirrors 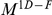. However, consideration of AP patterning in three dimensions allows us to address the experimentally observed DV-asymmetry in maternal Bcd and downstream AP morphogens. The inclusion of a DV-asymmetric Bcd signal led to qualitatively different patterning with 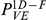 (**Fig. 4** **, columns 2,4**). This suggests that the assumption of DV and AP independence in previous modeling studies is violated. Parameter sensitivity remained high; parameter optimization made small changes to parameter values but led to significantly improved RMSE error (**Fig. 4** **, columns 4,5**).

Finally, two cases of DV model mismatch suggest modifications that could be incorporated into future models. First, anterior Gt is more highly expressed on the dorsal side of the embryo *in vivo*, but posterior Gt displays posterior-splaying. This expression localization is not accounted for by Bcd distribution alone and should be addressed in future models that also include input from the DV patterning system downstream of the active Dorsal protein distribution [65], [66]. Second, many protein species display DV-asymmetry in terms of anterior or posterior splaying. E.g., Cad bands anterior to the AP midline are anterior-splayed (**Fig. 4b** **, column 1**) while bands posterior to the AP midline are posterior-splayed. This pattern is observed for all modeled proteins (**Fig. 4** **, column 1**), though it is lacking in DV-symmetric 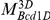 (**Fig. 4** **, column 2**). Addition of DV-symmetric Bcd (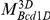) restores anterior-splaying aligned with the DV Bcd gradient (**Fig. 4** **, column 5**). This suggests that a missing posterior determinant may be responsible for posterior-splaying. The posterior maternal morphogen Nanos is a candidate that has not been included in previous models. With interpolated Cad and Tll, future models will explore the effects of posterior determinants such as Nanos [67] and, as examined in prior 1D models, Huckebein [37].

## Methods

### Model Construction

Building on the successful 1D/3D embryonic modeling approach of Umulis *et al*., [4], [68], we reimplemented the Jaeger *et al*. model of gap gene regulation (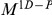) using the finite element method (FEM). This model represents six gene products as state variables: Cad, Gt, Hb, Kr, Kni, and Tll [13]. A seventh protein, Bcd (Bcd), is maintained at a constant concentration during gap gene patterning and is represented as a spatially heterogeneous stationary input [13], [63]. Each of the state variables is represented by a PDE,
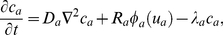
(1)where *c_a_* is the concentration of protein *a*, the first term on the right hand side represents diffusion, the second term represents gene expression, and the third term represents first order decay [13]. *D_a_* is the diffusion constant of protein *a* and λ*_a_* is the first order decay constant of protein *a*. *R_a_* is the maximal rate of gene expression of proteins *a* and *Φ_a_* is a sigmoid function,
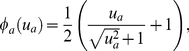
(2)which ranges from zero to one and accepts a regulatory argument *u_a_*:
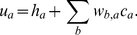
(3)Here, *h_a_* is a minimal regulatory threshold for expression, *w_b,a_* is an element in the regulatory matrix *W* representing the influence of protein *b* on the expression of protein *a* (ranging from −0.2 to 0.2), and *c_b_* is the local concentration of protein *b*. There are six PDEs representing protein proteins *a* = Cad, Gt, Hb, Kr, Kni, Tll (eqn. 1). In each PDE, the regulatory effects of all seven proteins, *b* = [41 Kr, Kni, Tll, Bcd], control protein expression (eqns. 2–3). PDEs are numerically solved using the FEM implemented in the software package COMSOL Multiphysics 3.5a [69]. Except for GRN parameters *w_b,a_*, these parameters are fixed at values in Jaeger *et al.* [13] and may be found in **Table S2**.

Note that previous 1D models were simulated by the spatially-discretized ordinary differential equations using the finite difference method: concentrations were tracked at uniformly-distributed nodes (nuclei) along the AP axis and diffusive fluxes across the Δ*x* inter-node distance were modeled as a first-order differential equations. As such, previously reported diffusion parameters (*Ď_a_*) were in units of inverse time [1/t]. To convert these parameters to diffusion constants (*D_a_*) with units of squared-length-per-time [L^2^/t], we multiplied *Ď_a_* by (Δ*x*)^2^. To compute Δ*x*, we took into account the length of the original model's domain (0.57 EL) and the number of nodes where the finite difference model was solved (58 nuclei). From these values, we approximated Δ*x* as 0.57EL/57. The model spans 0.35–0.92 or 0.57 EL and is divided into 57 intervals between 58 nodes. In the case of the 3D geometry, we further accounted for the curvature of the embryo in our approximation of Δ*x*. Scaling the embryo length to unity (1 EL), we observed an arc length of 1.14 along the lateral AP. Upon the assumption that curvature was uniformly-distributed along the AP axis, Δ*x* was computed as (0.57/1.14)EL/57. The approach slightly overestimates *D_a_* in the 3D model relative to 1D because most curvature occurs at the AP extrema and not the trunk, but this does not translate to a large impact on AP patterning versus 1D. Whereas finite difference models explicitly modify *Ď_a_* values to account for mitotic nuclear division and the halving of Δ*x*, the continuous FEM representation renders diffusion constants independent of nuclear density. It should be noted that this representation does not account for reduced effective diffusivity due to increased nuclear trapping. While nuclear density has been linked with decreased effective diffusivity in some simulations of Bcd diffusion [70], Grimm and Wieschaus found that transcription factor distributions are largely independent of nuclear density [71]. 3D nuclear density distributions have been published [47] and nuclear density-dependent diffusion is an area for further investigation.

We developed two FEM meshes on which to simulate spatiotemporal gap gene evolution. A 1D linear domain represents the 35–92% AP axis, and replicates the domain used in previous models [13]. By scaling diffusion constants and choosing initial conditions, the 1D domain also represents the 0–100% AP length (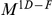). A 3D mesh modified from the VE geometry represents a realistic embryonic geometry. Though the embryonic syncytium includes the yolk interior of the embryo, nuclei are located within the periplasmic domain of the exterior surface [10], [49]. Cytoplasmic viscosity increases in the embryonic interior and is presumed to limit effective diffusion of gap gene products to the 2D layer in the periplasmic volume containing the nuclei. While some gap gene products may diffuse into yolk, this process may be considered as part of the decay terms, λ*_a_*. We took this into account when constructing the 3D domain. The reaction-diffusion equations (eqns. 1–3) are implemented as weak form PDEs on a 2D manifold (**Fig. S1**); this manifold is “wrapped” around the 3D embryonic geometry in 3D model implementations(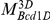,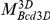,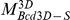).

Though the 3D domain is a closed surface without AP flux boundaries, the partial (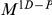) and full (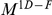) 1D domains are bounded at both termini by zero-flux conditions. These internal boundaries are unrealistic in the case of the partial AP length domain as there are no such physical barriers in the embryo; they were introduced in previous gap gene models to help account for artifacts in previously inferred GRNs [14], [42]–[44]. In full length 1D models the anterior and posterior ends of the embryo are realistically represented by zero-flux boundaries.

Numerical integration of PDEs requires specification of initial conditions as well as boundary conditions. For purposes of model comparison, we chose initial conditions specified in previous models [13]. On both 1D and 3D domains, the proteins Gt, Kni, Kr, and Tll have initial uniform concentrations of zero. Jaeger *et al*. provide initial non-uniform 1D distributions for Cad and Hb (**Fig. 3a**) [13]. These distributions span the entire AP length and provide initial conditions for both the partial and full length domains. Jaeger *et al.* also provide a constant exponential 1D Bcd distribution for the full AP length. These 1D distributions were used as initial conditions in the 1D models (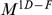 and 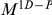). They were projected onto the 3D domain to approximate full 3D initial conditions (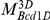, **Fig. 3b–d**). This projection was performed using built-in interpolation tools in the Comsol package. Provided AP-coordinates and corresponding concentration values, Comsol created a linear interpolant of DV-symmetric concentration values along the AP-axis of the 3D geometry.

While the Bcd data provided by Jaeger *et al.* describes the lateral AP distribution of Bcd, it fails to capture the observed DV asymmetry found in embryonic Bcd. Though sufficient for a 1D model (**Fig. 3a**), the resulting 3D distribution (**Fig. 3b**) qualitatively disagrees with VE data (**Fig. 3e**). We therefore built an interpolating function from the VE Bcd data and used this interpolant when simulating the model (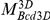). Again, we used Comsol's interpolation functionality. However, this interpolant required full 3D specification of coordinates. We used the coordinates of nuclei and corresponding Bcd concentration values provided in the VE. Because the software does not support interpolation on a 2D boundary (the periplasmic space) in a 3D geometry, we used nearest-neighbor interpolation (**Fig. 3e**). Because this Bcd distribution is represented in the model as a static interpolant, noise in the data (and hence the interpolant) is not smoothed by diffusion and decay. Initial attempts at directly importing VE Bcd data resulted in persistent asymmetries and mottled distributions inconsistent with data (**Fig. 4** **, column 3**). In an ideal situation, inter-embryo variability would be averaged out of VE data. However, the data set was generated with few replicates (13 embryos for Bcd [49]) and spatial noise remained. To remove this noise from the interpolant, we first fit a steady-state source-diffusion-decay (SDD) model of Bcd production [18] to VE Bcd data on the 3D domain (**Fig. 4a** **, column 1**). Once we had obtained agreement between this regularized Bcd distribution and the data, we used the solution of the SDD model to create a new interpolant. This smoothed interpolant shown in **Figure 3f** and 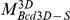
's output (**Fig. 4b–g** **, columns 4–5**) compares favorably with the results 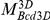 (**Fig. 4b–g** **, columns 3**).

Spatiotemporal regulation of gap gene expression spans the mitotic nuclear division between nuclear cycle 13 and 14a. For purposes of comparison, we chose to simulate the same time-course as previous models. We begin by simulating the conclusion of cycle 13 for sixteen minutes, mitosis for five minutes, and continue to simulate cycle 14a for the remaining forty-nine minutes [13]. The reaction-diffusion equations (eqn. 1–3) describe the model during interphase. During mitosis, gene expression (the second term in eqn. 1) is set to zero. Molecules may diffuse and decay, but they are not transcribed or translated while the chromatin is compacted for mitotic division.

This set of initial and boundary conditions, coupled with the reaction-diffusion equations and a geometric domain, constitutes a numerically soluble model. To calculate model error, we used a straightforward root mean squared error cost function:
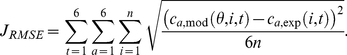
(5)Here, θ is the GRN parameter set, *n* is the number of data points in the 35%–92% EL region of the embryo, *a* is the index of protein species, *i* is the index of *n* nuclear coordinates, and *t* is the time index. This function sums the root squared error between model output from a given GRN, *c_a,mod_(*θ,*i,t)*, and experimental data, *c_x,exp_*(*i,t*), over data points *i*, model proteins *a*, and time *t*.


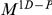
 was originally fit to immunofluorescence data in Jaeger *et al*. [13]. As a result, both the model's concentration units and GRN parameters are scaled to reflect observed relative intensity ranges of those data. To facilitate fitting between models utilizing Jaeger *et al.* 's parameters and VE data, we pre-scaled the VE data to agree with the initial conditions reported by Jaeger *et al.* This was performed by optimizing scaling factors *A_a_* and offsets *b_a_* such that the difference between Jaeger *et al.*'s initial conditions and the VE data was minimized,
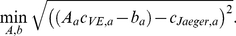
(6)The resulting scaling was applied to the VE data, allowing for direct comparison of model outputs. VE protein data is unavailable for Cad and Tll. For the former, we substituted expression data used by Jaeger *et al*. to fit the original model [13]. For the latter, we substituted Tll mRNA data from the VE and scaled it according to **eqn. 6**.

### Optimization

Using the cost function (eqn. 5), we optimized the full 1D and 3D models against scaled VE data using the Optimization Toolbox in MATLAB R2009a [72]. We began with local searches of the GRN weight matrix ***W*** (containing 42 parameters) using the constrained nonlinear minimization function fmincon(). We initialized these searches at the best-fit inferred GRN parameter set of the original modeling study and bounded all parameters within the interval [−0.2, 0.2] [13]. Parameter and cost function tolerances for stopping criteria were set to zero and the search was allowed to progress for 4200 model evaluations (100 evaluations per parameter), resulting in arrival at local minima. In the case of the DV-asymmetric Bcd model (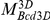), we subsequently included this locally optimal GRN in the initial population of a global search using genetic algorithms (GAs).

We used the GA as implemented in MATLAB. The population of size twenty genomes (parameter sets) was initialized with nineteen randomized parameter sets and the locally-optimized parameter set found for 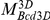. Stopping criteria were specified as a maximum of 100 generations or failure to improve cost function values above a tolerance of 10^−6^. The latter criterion increments a “stall” counter for each generation that fails to improve the score, ending the GA when the counter reaches fifty [72]. This algorithm was used to search the parameter space while fitting the 3D model incorporating DV-asymmetric Bcd (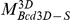).

## Supporting Information

Figure S1 **The VirtualEmbryo geometry.** A three-quarters view of the embryonic geometry with anterior (A), posterior (P), dorsal (D) and ventral (V) poles indicated.
(TIF)

Figure S2
**Scaled diffusion constants in DV-symmetric Bcd model** 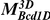 **.** The model is insensitive to small changes in the diffusion constant. (**A–G**) Lateral view of VE geometry is shown in rows **A–G** (Gt, Hb, Kni, Kr, Tll at *t* = 70 min, Cad at *t* = 56 min); Column 1 displays output from 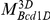 evaluated with GRN 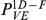 and diffusion constants *D_a_* scaled by 0.1; Column 2 displays output from 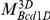 evaluated with GRN 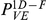 and diffusion constants *D_a_* scaled by 0.5; Column 3 displays output from 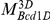 evaluated with GRN 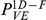 and diffusion constants *D_a_* scaled by 1; Column 4 displays output from 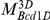 evaluated with GRN 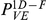 and diffusion constants *D_a_* scaled by 2; Column 5 displays output from 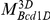 evaluated with GRN 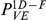 and diffusion constants *D_a_* scaled by 10.(TIF)

Figure S3
**Scaled diffusion constants in DV-asymmetric Bcd model** 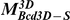 **.** The model is insensitive to small changes in the diffusion constant. (**A–G**) Lateral view of VE geometry is shown in rows **A–G** (Gt, Hb, Kni, Kr, Tll at *t* = 70 min, Cad at *t* = 56 min); Column 1 displays output from 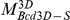 evaluated with GRN 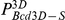 and diffusion constants *D_a_* scaled by 0.1; Column displays output from 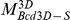 evaluated with GRN 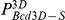 and diffusion constants *D_a_* scaled by 0.5; Column 3 displays output from 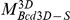 evaluated with GRN 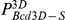 and diffusion constants *D_a_* scaled by 1; Column 4 displays output from 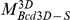 evaluated with GRN 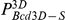 and diffusion constants *D_a_* scaled by 2; Column 5 displays output from 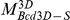 evaluated with GRN 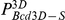 and diffusion constants *D_a_* scaled by 10.(TIF)

Movie S1
**Animated Cad pattern formation in the DV-symmetric Bcd model.** 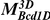 **.** Model output for Cad evaluated at the parameter set 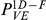. The video spans *t* = 0–70 min. Concentration colormap is identical to Fig. 4 and spans 0–250 concentration units.(M4V)

Movie S2
**Animated Gt pattern formation in the DV-symmetric Bcd model.** 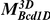 **.** Model output for Gt evaluated at the parameter set 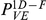. The video spans *t* = 0–70 min. Concentration colormap is identical to Fig. 4 and spans 0–250 concentration units.(M4V)

Movie S3
**Animated Hb pattern formation in the DV-symmetric Bcd model.** 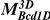 **.** Model output for Hb evaluated at the parameter set 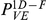. The video spans *t* = 0–70 min. Concentration colormap is identical to Fig. 4 and spans 0–250 concentration units.(M4V)

Movie S4
**Animated Kni pattern formation in the DV-symmetric Bcd model.** 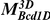 **.** Model output for Kni evaluated at the parameter set 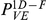. The video spans *t* = 0–70 min. Concentration colormap is identical to Fig. 4 and spans 0–250 concentration units.(M4V)

Movie S5
**Animated Kr pattern formation in the DV-symmetric Bcd model.** 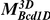 **.** Model output for Kr evaluated at the parameter set 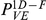. The video spans *t* = 0–70 min. Concentration colormap is identical to Fig. 4 and spans 0–250 concentration units.(M4V)

Movie S6
**Animated Tll pattern formation in the DV-symmetric Bcd model.** 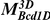 **.** Model output for Tll evaluated at the parameter set 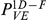. The video spans *t* = 0–70 min. Concentration colormap is identical to Fig. 4 and spans 0–250 concentration units.(M4V)

Movie S7
**Animated Cad pattern formation in the DV-asymmetric Bcd model.** 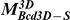 **.** Model output for Cad evaluated at the parameter set 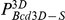. The video spans *t* = 0–70 min. Concentration colormap is identical to Fig. 4 and spans 0–250 concentration units.(M4V)

Movie S8
**Animated Gt pattern formation in the DV-asymmetric Bcd model.** 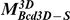 **.** Model output for Gt evaluated at the parameter set 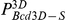. The video spans *t* = 0–70 min. Concentration colormap is identical to Fig. 4 and spans 0–250 concentration units.(M4V)

Movie S9
**Animated Hb pattern formation in the DV-asymmetric Bcd model.** 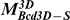 **.** Model output for Hb evaluated at the parameter set 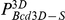. The video spans *t* = 0–70 min. Concentration colormap is identical to Fig. 4 and spans 0–250 concentration units.(M4V)

Movie S10
**Animated Kni pattern formation in the DV-asymmetric Bcd model.** 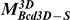 **.** Model output for Kni evaluated at the parameter set 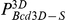. The video spans *t* = 0–70 min. Concentration colormap is identical to Fig. 4 and spans 0–250 concentration units.(M4V)

Movie S11
**Animated Kr pattern formation in the DV-asymmetric Bcd model.** 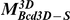 **.** Model output for Kr evaluated at the parameter set 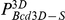. The video spans *t* = 0–70 min. Concentration colormap is identical to Fig. 4 and spans 0–250 concentration units.(M4V)

Movie S12
**Animated Tll pattern formation in the DV-asymmetric Bcd model.** 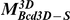 **.** Model output for Tll evaluated at the parameter set 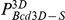. The video spans *t* = 0–70 min. Concentration colormap is identical to Fig. 4 and spans 0–250 concentration units.(M4V)

File S1 **Mesh coordinates for the VirtualEmbryo.** Matlab-accessible file containing indexed vertex coordinates and relations defining the triangular elements of the mesh in Figure S1.
(MAT)

Table S1 **GRN Parameter Values.**
(DOC)

Table S2 **Non-GRN Parameters (Unoptimized).**
(DOC)
