# Supplementary figures and images for: Analysis of Gap Gene Regulation in a 3D Organism-Scale Model of the Drosophila melanogaster Embryo
Source: PLoS One. 2011 Nov 16;6(11):e26797. doi: 10.1371/journal.pone.0026797 (PMC3217930; doi:10.1371/journal.pone.0026797)

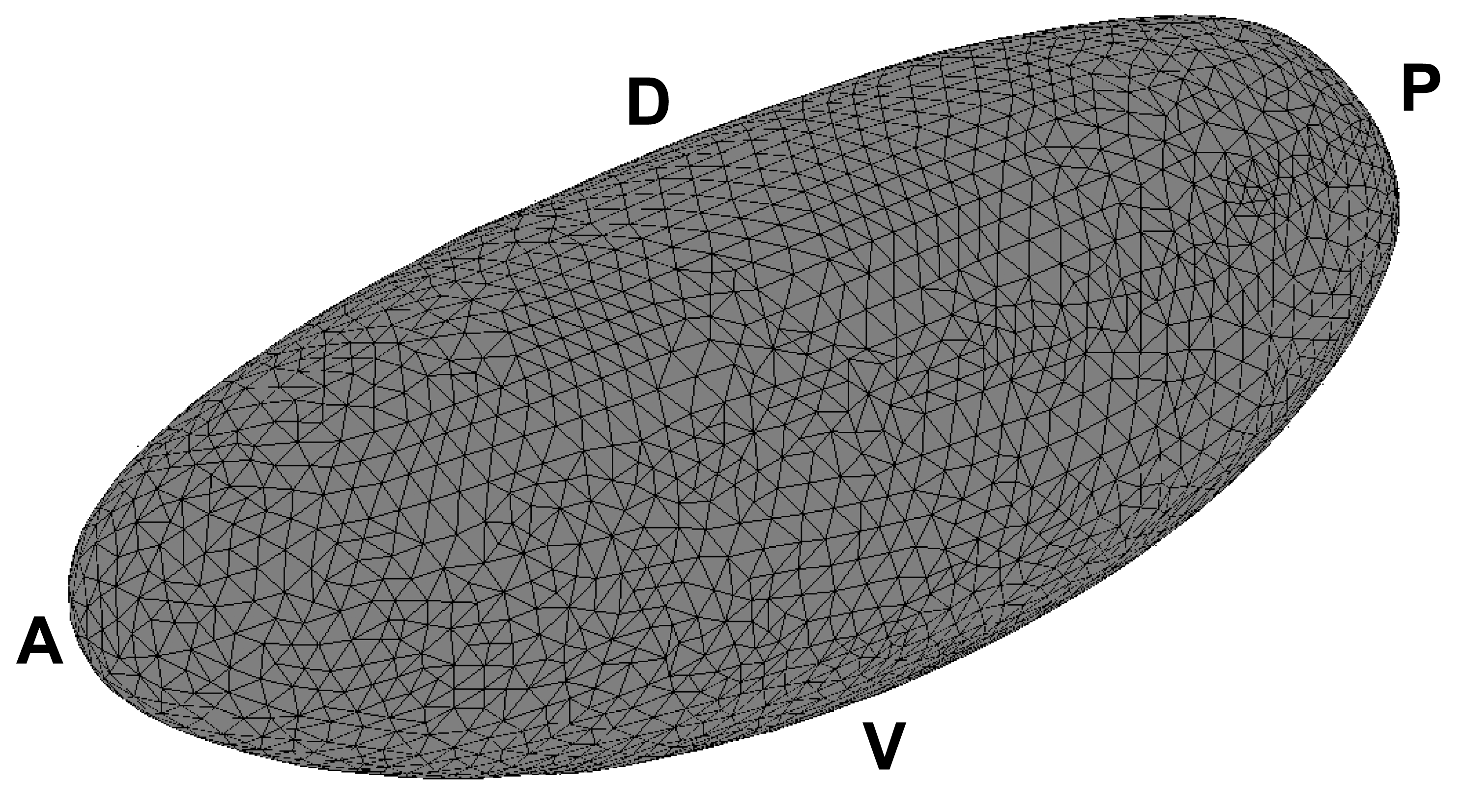

Supplement: Figure S1 — The VirtualEmbryo geometry. A three-quarters view of the embryonic geometry with anterior (A), posterior (P), dorsal (D) and ventral (V) poles indicated. (TIF) [file pone.0026797.s001.tif]

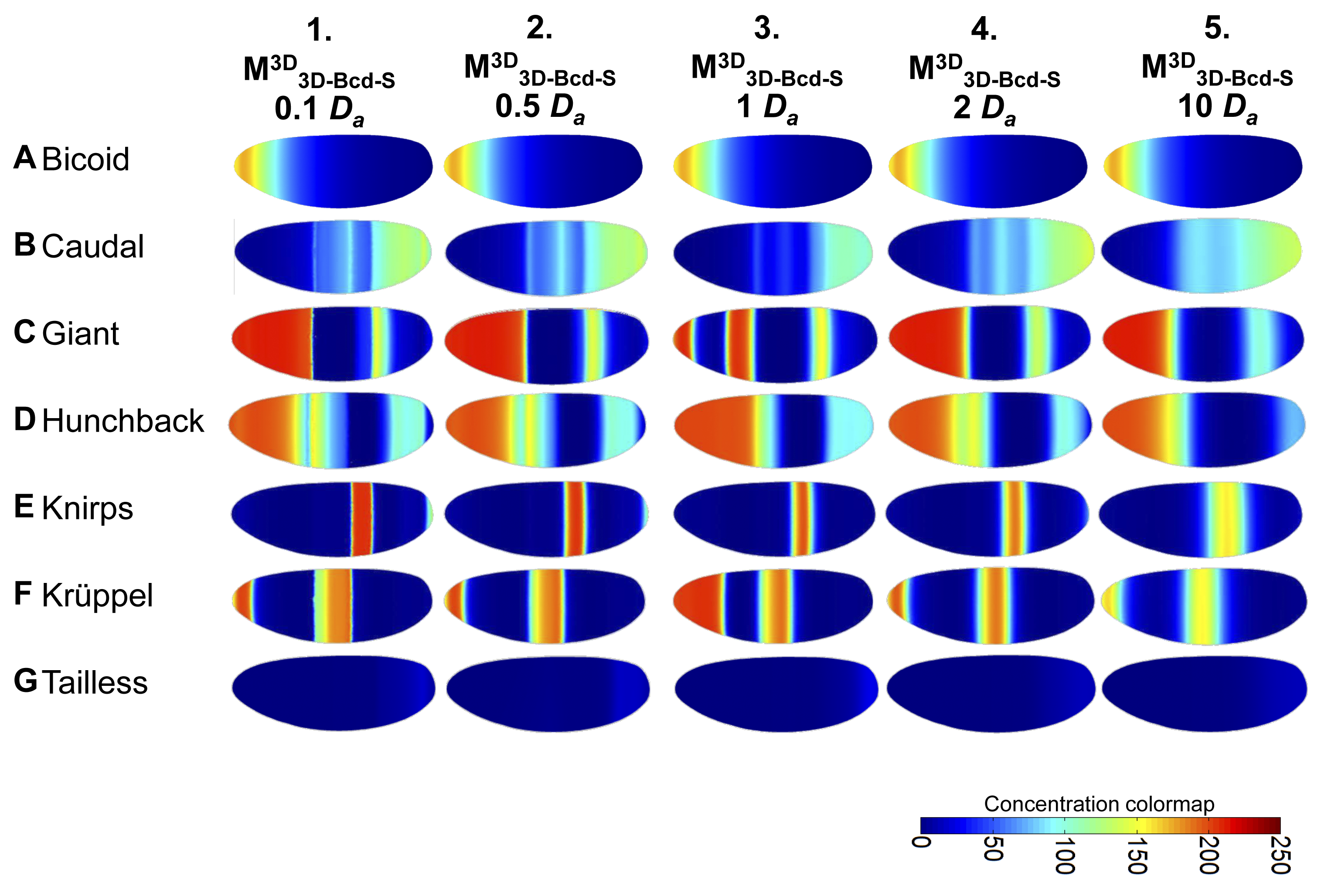

Supplement: Figure S2 — Scaled diffusion constants in DV-symmetric Bcd model . The model is insensitive to small changes in the diffusion constant. (A–G) Lateral view of VE geometry is shown in rows A–G (Gt, Hb, Kni, Kr, Tll at t = 70 min, Cad at t = 56 min); Column 1 displays output from evaluated with GRN and diffusion constants Da scaled by 0.1; Column 2 displays output from evaluated with GRN and diffusion constants Da scaled by 0.5; Column 3 displays output from evaluated with GRN and diffusion constants Da scaled by 1; Column 4 displays output from evaluated with GRN and diffusion constants Da scaled by 2; Column 5 displays output from evaluated with GRN and diffusion constants Da scaled by 10. (TIF) [file pone.0026797.s002.tif]

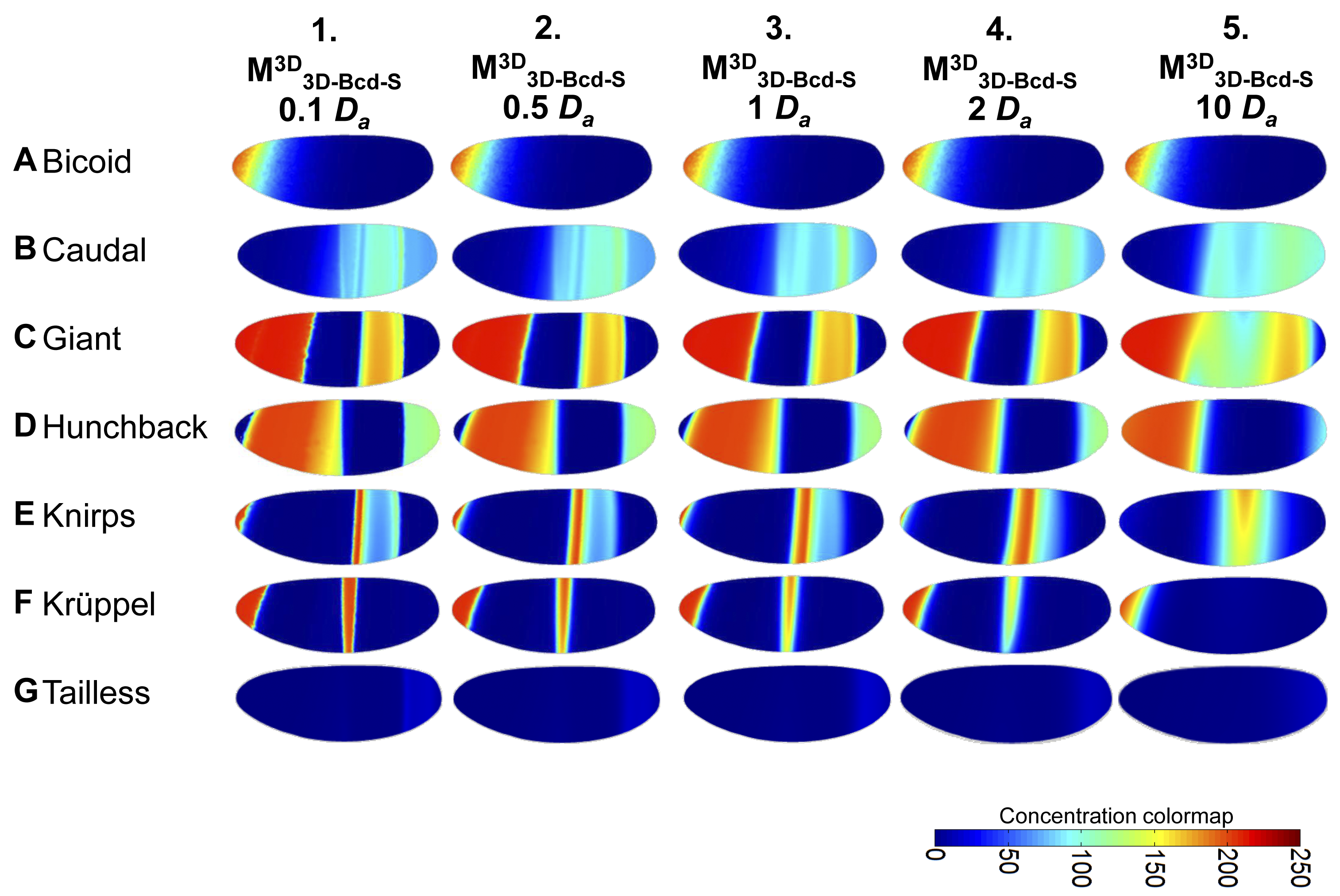

Supplement: Figure S3 — Scaled diffusion constants in DV-asymmetric Bcd model . The model is insensitive to small changes in the diffusion constant. (A–G) Lateral view of VE geometry is shown in rows A–G (Gt, Hb, Kni, Kr, Tll at t = 70 min, Cad at t = 56 min); Column 1 displays output from evaluated with GRN and diffusion constants Da scaled by 0.1; Column displays output from evaluated with GRN and diffusion constants Da scaled by 0.5; Column 3 displays output from evaluated with GRN and diffusion constants Da scaled by 1; Column 4 displays output from evaluated with GRN and diffusion constants Da scaled by 2; Column 5 displays output from evaluated with GRN and diffusion constants Da scaled by 10. (TIF) [file pone.0026797.s003.tif]
