# Supplementary material for: Analysis of Gap Gene Regulation in a 3D Organism-Scale Model of the Drosophila melanogaster Embryo
Source: PLoS One. 2011 Nov 16;6(11):e26797. doi: 10.1371/journal.pone.0026797 (PMC3217930; doi:10.1371/journal.pone.0026797)
Supplement: Table S1 — GRN Parameter Values. (DOC) [file pone.0026797.s017.doc]

| **Table S1: GRN Parameter Values** | | | | | | | |
| --- | --- | --- | --- | --- | --- | --- | --- |
| W_b🡨a_ (conc^-1^) | $P^{0}$ | $P_{1D-P}^{1D-F}$ | $P_{VE}^{1D-F}$ | $P_{Bcd1D}^{3D}$ | $P_{Bcd3D}^{3D}$ | $P_{Bcd3D-S}^{3D}$ | σ_P_ |
| Cad 🡨 Cad | -0.068 | -0.068 | -0.05545 | -0.06739 | -0.06753 | -0.06724 | 0.004983 |
| Cad 🡨 Hb | -0.073 | -0.0726 | -0.04316 | -0.07177 | -0.07264 | -0.07155 | 0.011914 |
| Cad 🡨 Kr | -0.05 | -0.05008 | -0.04001 | -0.04992 | -0.04992 | -0.03952 | 0.005278 |
| Cad 🡨 Gt | -0.056 | -0.0563 | -0.05241 | -0.05596 | -0.05596 | -0.02386 | 0.012929 |
| Cad 🡨 Kni | -0.038 | -0.03813 | -0.0464 | -0.03615 | -0.03752 | -0.03611 | 0.003865 |
| Cad 🡨 Tll | -0.034 | -0.034 | -0.08397 | -0.03375 | -0.03393 | -0.03144 | 0.020659 |
| Cad 🡨 Bcd | -0.04 | -0.04 | -0.01014 | -0.0418 | -0.01226 | -0.01934 | 0.014946 |
| Hb🡨 Cad | 0.022 | 0.022515 | 0.024482 | 0.022383 | 0.022383 | 0.022382 | 0.000895 |
| Hb 🡨 Hb | 0.019 | 0.019171 | 0.008043 | 0.018707 | 0.018707 | 0.018086 | 0.00438 |
| Hb 🡨 Kr | 0.001 | 0.000819 | 0.002504 | 0.001407 | 0.001407 | 0.00196 | 0.000624 |
| Hb 🡨 Gt | 0.011 | 0.011081 | -0.00169 | 0.010731 | 0.011219 | 0.011462 | 0.005227 |
| Hb 🡨 Kni | -0.166 | -0.16401 | -0.12591 | -0.11875 | -0.16562 | -0.12985 | 0.022408 |
| Hb 🡨 Tll | 0.003 | 0.002798 | 0.02075 | 0.003135 | 0.003135 | 0.003957 | 0.007174 |
| Hb 🡨 Bcd | 0.05 | 0.050158 | 0.05526 | 0.050267 | 0.015583 | 0.062363 | 0.016251 |
| Kr🡨 Cad | 0.033 | 0.033083 | 0.031761 | 0.033324 | 0.03318 | 0.033502 | 0.000621 |
| Kr 🡨 H | -0.014 | -0.01408 | -0.01706 | -0.01372 | -0.01379 | -0.01248 | 0.001523 |
| Kr 🡨 Kr | 0.017 | 0.016974 | 0.007245 | 0.016765 | 0.016765 | 0.017671 | 0.004011 |
| Kr 🡨 Gt | -0.076 | -0.07637 | -0.07307 | -0.07523 | -0.07566 | -0.0717 | 0.001862 |
| Kr 🡨 Kni | -0.015 | -0.01499 | -0.0123 | -0.01533 | -0.01533 | -0.01606 | 0.001301 |
| Kr 🡨Tll | -0.08 | -0.0795 | -0.06905 | -0.07997 | -0.07997 | -0.05334 | 0.010845 |
| Kr🡨 Bcd | 0.129 | 0.128978 | 0.130053 | 0.128792 | 0.039917 | 0.131845 | 0.036685 |
| Gt 🡨 Cad | 0.029 | 0.029012 | 0.028068 | 0.029179 | 0.029179 | 0.030979 | 0.00095 |
| Gt 🡨 Hb | -0.018 | -0.01782 | -0.0203 | -0.01842 | -0.01842 | -0.01774 | 0.000952 |
| Gt 🡨 Kr | -0.11 | -0.11 | -0.10785 | -0.11008 | -0.11011 | -0.10661 | 0.001507 |
| Gt 🡨 Gt | 0.011 | 0.011179 | 0.003018 | 0.011051 | 0.010633 | 0.020263 | 0.005467 |
| Gt 🡨 Kni | -0.001 | -0.00089 | 0.00017 | -0.00127 | -0.00127 | 0.003246 | 0.001756 |
| Gt 🡨 Tll | -0.02 | -0.0203 | 0.001364 | -0.01979 | -0.01967 | -0.01808 | 0.00858 |
| Gt 🡨 Bcd | 0.177 | 0.176424 | 0.181027 | 0.177275 | 0.054792 | 0.179195 | 0.050403 |
| Kni🡨 Cad | 0.037 | 0.037184 | 0.032213 | 0.037009 | 0.036893 | 0.037298 | 0.001991 |
| Kni 🡨 Hb | -0.027 | -0.02758 | -0.00752 | -0.02298 | -0.02689 | -0.02571 | 0.007734 |
| Kni 🡨 Kr | -0.024 | -0.02432 | -0.03323 | -0.02353 | -0.02353 | -0.02332 | 0.003891 |
| Kni 🡨 Gt | -0.09 | -0.08995 | -0.07284 | -0.08915 | -0.08952 | -0.08785 | 0.006763 |
| Kni 🡨 Kni | 0.045 | 0.044792 | 0.045294 | 0.045337 | 0.045337 | 0.045792 | 0.000341 |
| Kni 🡨 Tll | -0.077 | -0.07445 | -0.04179 | -0.07613 | -0.07696 | -0.07558 | 0.014008 |
| Kni 🡨 Bcd | 0.097 | 0.097206 | 0.071687 | 0.096979 | 0.030063 | 0.1025 | 0.027938 |
| Tll🡨 Cad | -0.018 | -0.01776 | -0.02742 | -0.01776 | -0.01776 | -0.01648 | 0.004065 |
| Tll🡨 HB | -0.106 | -0.10636 | -0.10476 | -0.1063 | -0.10648 | -0.09852 | 0.00311 |
| Tll🡨 Kr | -0.106 | -0.1062 | -0.11634 | -0.10577 | -0.10585 | -0.10552 | 0.004281 |
| Tll🡨 Gt | -0.082 | -0.082 | -0.05158 | -0.08187 | -0.08187 | -0.08213 | 0.012409 |
| Tll🡨 Kni | -0.137 | -0.12525 | -0.118 | -0.13695 | -0.13695 | -0.13407 | 0.007968 |
| Tll🡨 Tll | -0.003 | -0.00299 | 0.045177 | -0.00254 | -0.00261 | 0.001568 | 0.019305 |
| Tll🡨 Bcd | -0.007 | -0.00698 | -0.01495 | -0.0069 | -0.00214 | -0.00478 | 0.004282 |
