# Supplementary material for: Analysis of Gap Gene Regulation in a 3D Organism-Scale Model of the Drosophila melanogaster Embryo
Source: PLoS One. 2011 Nov 16;6(11):e26797. doi: 10.1371/journal.pone.0026797 (PMC3217930; doi:10.1371/journal.pone.0026797)
Supplement: Table S2 — Non-GRN Parameters (Unoptimized). (DOC) [file pone.0026797.s018.doc]

| **Table S2: Non-GRN Parameters (Unoptimized)** | | |
| --- | --- | --- |
| Parameter* | 1D Model Values | 3D Model Values |
| D_Cad_ (EL^2^ min^-1^) | 2 × 10^-5^ | 1.53 × 10^-5^ |
| D_Gt_ (EL^2^ min^-1^) | 1.42 × 10^-5^ | 1.1 × 10^-5^ |
| D_Hb_ (EL^2^ min^-1^) | 2 × 10^-5^ | 1.53 × 10^-5^ |
| D_Kni_ (EL^2^ min^-1^) | 2 × 10^-5^ | 1.53 × 10^-5^ |
| D_Kr_ (EL^2^ min^-1^) | 2 × 10^-5^ | 1.53 × 10^-5^ |
| D_Tll_ (EL^2^ min^-1^) | 2 × 10^-5^ | 1.53 × 10^-5^ |
| R_Cad_ (conc min^-1^) | 20 | 20 |
| R_Gt_ (conc min^-1^) | 15.79 | 15.79 |
| R_Hb_ (conc min^-1^) | 19.61 | 19.61 |
| R_Kni_ (conc min^-1^) | 12.18 | 12.18 |
| R_Kr_ (conc min^-1^) | 16.37 | 16.37 |
| R_Tll_ (conc min^-1^) | 11.91 | 11.91 |
| h_Cad_ | 13.46 | 13.46 |
| h_Gt_ | -3.5 | -3.5 |
| h_Hb_ | -3.5 | -3.5 |
| h_Kni_ | -3.5 | -3.5 |
| h_Kr_ | -3.5 | -3.5 |
| h_Tll_ | 8.1735 | 8.1735 |
| λ_Cad_ (min^-1^) | log(2)/18 | log(2)/18 |
| λ_Gt_ (min^-1^) | log(2)/9.577 | log(2)/9.577 |
| λ_Hb_ (min^-1^) | log(2)/7.254 | log(2)/7.254 |
| λ_Kni_ (min^-1^) | log(2)/12.499 | log(2)/12.499 |
| λ_Kr_ (min^-1^) | log(2)/8.98 | log(2)/8.98 |
| λ_Tll_ (min^-1^) | log(2)/16.82 | log(2)/16.82 |

*EL = Embryo Length; conc = scaled concentration units
